# Variation in Annual Volume at a University Hospital Does Not Predict Mortality for Pancreatic Resections

**DOI:** 10.1155/2008/190914

**Published:** 2009-02-25

**Authors:** Rita A. Mukhtar, Omar M. Kattan, Hobart W. Harris

**Affiliations:** Department of Surgery, University of California, San Francisco, CA 94143-0104, USA

## Abstract

Annual volume of pancreatic resections has been shown to affect mortality rates, prompting recommendations to regionalize these procedures to high-volume hospitals. Implementation has been difficult, given the paucity of high-volume centers and the logistical hardships facing patients. Some studies have shown that low-volume hospitals achieve good outcomes as well, suggesting that other factors are involved. We sought to determine whether variations in annual volume affected patient outcomes in 511 patients who underwent pancreatic resections at the University of California, San Francisco between 1990 and 2005. We compared postoperative mortality and complication rates between low, medium, or high volume years, designated by the number of resections performed, adjusting for patient characteristics. Postoperative mortality rates did not differ between high volume years and medium/low volume years. As annual hospital volume of pancreatic resections may not predict outcome, identification of actual predictive factors may allow low-volume centers to achieve excellent outcomes.

## 1. INTRODUCTION

The desire to improve surgical outcomes has resulted in significant interest in volume-outcome relationships. As insurance companies continue developing policies that may use hospital volume to influence patient choice of treatment center [1], the importance of the issue increases. For pancreatic resections specifically, many studies have found that higher annual volume predicts lower mortality rates [1–10]. Such findings have led some to advocate regionalization of pancreatic resections [3, 7–11]. Despite this call, however, there have been practical limitations to such centralization [12]. In addition, the validity of studies that typically rely on administrative databases for information has been questioned [13]. A recent study from our institution suggested that the observed benefits at high-volume hospitals may be “exported” to low-volume hospitals, suggesting that factors other than volume alone may contribute to outcomes [14].

Few studies have focused on the effects of annual fluctuations in hospital volume on outcome for pancreatic resections. Additionally, most studies group different types of pancreatic resections together instead of distinguishing between pancreatic head resections and non-head resections [10]. Furthermore, many studies have examined the relationship between annual volume and mortality but have not addressed whether volume affects postoperative complication rates. We sought to determine whether the reported volume-outcome relationships would be observed over time at a single institution for both mortality and postoperative complications, and whether those relationships would hold true for specific types of pancreatic resections. We also looked at trends in pancreatic resections performed at our institution over 16 years to determine which factors were associated with postoperative complications.

## 2. MATERIALS AND METHODS

### 2.1. Patient selection

The paper and electronic charts of 511 patients who underwent pancreatic resection at UCSF's Moffitt Hospital between January 1990 and October 2005 were retrospectively reviewed. Patients were identified through a computerized search for procedural codes from the *International Classification of Diseases, 9th Revision, Clinical Modification (ICD-9-CM)* corresponding to the following diagnoses: partial pancreatectomy, proximal pancreatectomy, distal pancreatectomy, radical subtotal pancreatectomy, other partial pancreatectomy, and pancreaticoduodenectomy (PD). We excluded patients who were identified by the computerized search but did not in fact undergo actual pancreatic resection (e.g., those who had pancreaticojejunostomy, pancreatic debridement, or pancreas transplant). We also excluded patients who were younger than 16 years of age at the time of resection. Race/ethnicity was collected as recorded on the patients' records. The University of California, San Francisco Committee on Human Research approved this study prior to the review of patient records.

### 2.2. Demographic and clinical characteristics

The following data were recorded for each patient: age, gender, ethnicity, ASA score, indication for operation, operation type, pathologic diagnosis, postoperative complications, length of stay, and in-hospital mortality.

Operation types included PD, pylorus-preserving PD, distal pancreatectomy, spleen-sparing distal pancreatectomy, subtotal pancreatectomy, total pancreatectomy, and total pancreatectomy with islet autotransplantation.

Pathologic diagnoses were grouped into pancreatic adenocarcinoma, periampullary tumor, cystic tumor, neuroendocrine tumor, chronic pancreatitis, gastrointestinal stromal tumor, metastatic tumors, or “other.” Periampullary tumors included neoplasms of the common bile duct, ampulla of Vater, and duodenum. Cystic tumors included intraductal papillary mucinous tumors, mucinous cystadenoma, mucinous cystadenocarcinoma, microcystic adenoma, serous cystadenoma, and solid and papillary neoplasms. All other findings were categorized as “other.”

### 2.3. Outcomes

Postoperative complications were assessed by clinical diagnosis noted in the medical record, and included wound infection, pancreatic fistula/leak, gastroparesis, bile leak, reoperation, bleeding, pneumonia, and urinary tract infection (UTI). Less frequent complications were grouped together as “other,” a category that included pleural effusion, pulmonary edema, pulmonary embolus, arrhythmia, myocardial infarction, wound dehiscence, *Clostridium difficile* colitis, acute renal failure, incisional hernia, and bacteremia.

To evaluate the effect of annual volume on outcomes, each study year was listed by number of resections performed. To maximize differences between the low volume and high volume groups, the years were then divided by volume into three roughly equally sized groups: high, medium, and low. To further determine whether the volume of a specific type of pancreatic resection could predict outcomes, years were categorized in two ways: based on volume of pancreatic head resections performed, and by the volume of non-head resections performed.

### 2.4. Statistical analysis

We used multiple logistic regression to examine all relationships between annual volume and outcome, adjusting for patient gender, presence of underlying malignancy on pathological diagnosis, race (white or nonwhite), and age. Additionally, *t*-tests and chi-square tests were used where appropriate, with statistical significance set at *P* < .05. Data are presented as mean ± standard deviation (SD) unless otherwise indicated. Data were analyzed using statistical analysis software (SAS) (Cary, NC, USA) and STATA (College Station, Tex, USA).

## 3. RESULTS

### 3.1. Patient characteristics

Of the 511 patients who underwent pancreatic resections during the 16-year study period, 52% were females.The average age was 58.6 ± 14.7 years. Most patients (61%) are self-identified as White.

### 3.2. Procedures

The majority (60%) of resections performed were pancreatic head resections (PD); non-head resections (40%) comprised distal, subtotal, or total pancreatectomies. Among the pancreatic head resections, more than (60%) were pylorus-preserving PD as opposed to classical PD.

Among the non-head resections, 85% were distal pancreatectomies.

### 3.3. Pathologic diagnoses

The most common pathologic diagnosis was adenocarcinoma of the pancreas (25%), followed by periampullary tumors and cystic tumors. Three of the 33 patients with a preoperative diagnosis of chronic pancreatitis had final pathologic results that indicated malignancy. Conversely, 15 (24%) of the 62 patients with a final diagnosis of chronic pancreatitis were erroneously believed to have a malignancy preoperatively.

### 3.4. Comparison of complication rates for head and non-head pancreatic resections

Rates of wound infections, intraabdominal abscesses, pancreatic fistulas, gastroparesis, reoperations, and “other” complications were significantly higher in patients who underwent pancreatic head resections than in those who underwent non-head resections. Not surprisingly, patients who underwent pancreatic head resections also had significantly longer hospital stays (mean of 14.9 days versus 10.7 days). Length of stay ranged from 2–373 days with a median of 11 days for pancreatic head resections, and 1–56 days with a median of 8 days for non-head resections. Additionally, slightly more surgical complications occurred in patients with an underlying malignancy (34.4%) than in patients with benign disease (25.3%).

### 3.5. Effect of annual volume on resection outcome

**Figure 1:**
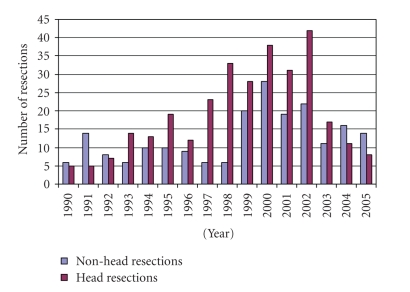
*Annual pancreatic resection volume broken down by head resections and non-head resections*. Trends in the volume of non-head pancreatic resections generally followed those of head resections, with some exceptions.

An average of 32 ± 17 pancreatic resections were done annually during the years included in this study. A higher than average number of resections (*n* = 262) was performed between 1998–2002, but the numbers declined beginning in 2003, possibly related to the departure of one particular surgeon. With some exceptions, trends in the volume of non-head pancreatic resections generally followed those of head resections (Figure 1). However, because the volume of head resections did not necessarily increase or decrease concordantly with the volume of non-head resections, we first used the rates of pancreatic *head* resections to define high, medium, and low volume years, and then used the rates of *non-head* resections to do so (Table 1).

We found that among patients who underwent any pancreatic resection in low volume years as defined by number of head resections performed, there were no in-hospital deaths. When medium and low volume years were combined for purposes of statistical analysis, there was no difference in death rates for either pancreatic head resections (odds ratio = 1.62, *P* = .64) or non-head resections (odds ratio = 1.09, *P* = .9) between high volume years and low/medium volume years. In addition, there were no significant differences in postoperative complication rates among patients undergoing non-head resection in high versus low volume years. The incidence of gastroparesis (odds ratio = 0.57, *P* = .7) and bleeding (odds ratio = 0.344, *P* = .398) was lower in high volume years, but not significantly so, and the incidence of UTIs was higher, but again, not significantly so (odds ratio = 4.26, pq = 0.199) (Table 2). The only difference was that there were significantly fewer bile leaks in patients who underwent pancreatic head resection in a high-volume head resection year (odds ratio = 0.09, *P* < .05) (Table 2).

When the rates of pancreatic non-head resections were used to define high, medium, and low volume years, there was no significant difference in death rates for either pancreatic head resections (odds ratio = 1.13, *P* = .923) or non-head resections (odds ratio = 0.652, *P* = .611) performed in high volume years versus low volume years. The same was true for complication rates (odds ratios = 0.734 for non-head resections, and 0.798 for head resections). Although for patients undergoing non-head resections, the incidence of gastroparesis in high volume years was lower, the difference was not significant (odds ratio = 0.338, *P* = .453), nor was the higher incidence of intraabdominal abscesses (odds ratio = 1.92, *P* = .554). Similarly, patients undergoing head resections had fewer wound infections, bile leaks, cases of pneumonia, and less bleeding in high volume years, but again, the differences were not significant (Table 3).

## 4. DISCUSSION

Our findings show that at a single institution variation in annual pancreatic resection volume does not affect mortality. In fact, there were no in-hospital deaths following pancreatic head resection during the six low volume years included in the study. Although our hospital's volumes may be considered high by some criteria, during four of the six low volume years, we did not meet the Leapfrog consortium's cutoff for being an index center (11 pancreatic head resections per year) [15], suggesting that our low volume group is truly reflective of low volumes based on nationally accepted standards. The results presented here are discordant with the previously demonstrated inverse relationship between pancreatic resection volume and mortality [2–10]. In fact, the findings seem to suggest that “once a high-volume hospital, always a high-volume hospital,” even if fluctuations in volume actually give that hospital low-volume status for a given period of time. If this is the case, there must be some factor that affords the hospital the ability to maintain excellent mortality rates independent of volume. The existence of such a factor supports the notion that the systems in place at a particular institution [4, 16] may be the effectors of outcome, and suggests that a higher volume may be a proxy for the presence of these systems. This may explain why some low-volume hospitals have been able to achieve low mortality rates [3], and why it may be possible to “export” good outcomes to low-volume hospitals [14]. Many studies support the idea that volume is only one part of a complex system of factors that affect outcomes, with findings such as race [17], the proportion of minorities treated at a given hospital, surgeon volume [18], and even surgeon age [19] playing roles in surgical mortality. An advantage of the grouping method used in our study is that each group contains a diverse range of years (e.g., the low volume group contains years 1990 and 2005), possibly reducing the contribution from any one surgeon.

Although some trends toward fewer complications in high volume years were observed, only the incidence of bile leaks following pancreatic head resection was significantly lower in the high volume years. We have not seen this specific finding in published reports, but a recent study did show decreased complication rates following pancreatic resection as volume increased in an already high-volume hospital [20]. Some have suggested that differences in the quality of managing postoperative complications may account for differences in-hospital outcomes [21]; however, in the prior study, and the one reported here, mortality rates remained unchanged despite changing trends in complication rates. Additionally, during the period of time included in the study, there was no system-wide initiation of a preoperative or postoperative pathway for patients undergoing pancreatic resection.

It is possible that although we had high and low volume years, we failed to find a difference in mortality rates because we divided pancreatic resections into head and non-head resections when creating our volume groups. Had we instead used all pancreatic resections to define the groups, our low volume years may have exceeded the volume designated as “low” in other studies. However, because many studies have grouped different types of pancreatic resections together [10], and to be consistent with a large study which showed a volume-outcome relationship for pancreatic head resections [3], we chose to split them up in order to see whether there was any specific predictive value for a given resection type. Additionally, we still had a clear difference in the average number of resections performed in high versus low volume years.

In our analysis of other factors that may contribute to morbidity and mortality, our results were mostly consistent with previously reported data, with patients undergoing pancreatic head resection experiencing more complications than those undergoing non-head resection [20]. In our series, 31.4% of patients had one or more surgical complications following pancreatic head resection, consistent with the reported overall morbidity of 30–55% [22]. In our study, the incidence of pancreatic fistula after pancreatic head resection was 10.5% and consistent with prior reports [23, 24], while the incidence following other pancreatic resections was 3.9%. Our findings suggest that the creation of the pancreatic anastomosis carries a higher risk of pancreatic leak than transection of the pancreas. Interestingly, others have reported a higher incidence of pancreatic fistulas with left-sided pancreatic resections [25].

For patients with malignant disease, the overall surgical complication rate was 34.4% compared to 25.3% for patients with benign disease. Although patients with malignant disease tended to have more complications, only the incidence of gastroparesis came close to differing significantly. This is fairly consistent with other studies which have shown no differences in surgical complications between these two patient groups [4]. The higher observed incidence of gastroparesis may be due to the higher incidence of gastroparesis reported with malignancy itself [26–29].

Our study has many important limitations. First, it was retrospective. A prospective study would have allowed more accurate assessment of postoperative complications, since we had relied on physician diagnosis and notation of complications in the medical record. As such, definitions of particular diagnoses were likely inconsistent across physicians and the time course of the study. Additionally, review of inpatient charts indicated in-hospital mortality, but we were not able to accurately assess deaths that may have occurred after discharge, limiting the inclusion of potential deaths due to late complications. Moreover, although we included over 500 patients in this study, the study is still limited to a single hospital's experience.

Clearly, many studies have observed lower mortality rates for pancreatic resections performed at high-volume hospitals. Understanding the precise mechanism behind this association may allow us to improve outcomes for patients facing diseases that require pancreatic resection. The fact that this relationship is not borne out at a single hospital that had fluctuations in volume over time, that race has been shown to affect outcome for some procedures irrespective of hospital volume [17], that clinical studies are less likely to find volume-outcome relationships than administrative studies [13], and that patient characteristics at high and low volume hospitals differ significantly [9, 12], all points to a much more complex explanation than volume alone. Of course, these data give no insight into how hospitals acquire the ability to provide good outcomes after pancreatic resections. Surgical volume may indeed play a role; however, annual volume alone does not appear to be an adequate predictor of postoperative mortality at our institution. Even if the answer were simply volume, we saw that implementing regionalization faces considerable obstacles [10, 12]. The goal then is to determine how to improve outcomes for patients who will continue to be treated at low-volume hospitals. Our findings suggest that excellent outcomes for pancreatic resections are possible despite changes in volume. Further investigation is needed into what elements make that possible.

## 5. CONCLUSIONS

Annual volume of pancreatic resections does not predict postoperative mortality at a major academic medical center. With the exception of a lower incidence of bile leaks following pancreatic head resections performed in high volume years, there are no differences in postoperative complications following pancreatic resections in high versus low volume years. These findings contradict previously published studies and suggest the need for further investigation into the predictors of outcomes following pancreatic resections.

## Figures and Tables

**Table 1:** High, medium, and low volume groups based on either pancreatic head resection volume, or non-head resection volume.

Head resection volume groups*	Number of head resections performed	Non-head resection volume groups	Number of non-head resections performed
High volume years		High volume years	

2002	42	2000	28
2000	38	2002	22
1998	33	1999	20
2001	31	2001	19
1999	28	2004	16

Medium volume years		Medium volume years	

1997	23	2005	14
1995	19	1991	14
2003	17	1994	10
1993	14	1995	10
1994	13	2003	11

Low volume years		Low volume years	

1996	12	1996	9
2004	11	1992	8
2005	8	1990	6
1992	7	1993	6
1991	5	1997	6
1990	5	1998	6

*Each study year was listed by number of resections performed. The years were then divided by volume into three roughly equally sized groups: high, medium, and low.

**Table 2:** Postoperative complication and mortality rates by low, medium, and high volume years as defined by the number of *pancreatic head resections* performed.*

	Low volume years*		Medium volume years		High volume years		
	Non-head resections (*n* = 66)	Head resections (*n* = 49)	Non-head resections (*n* = 43)	Head resections (*n* = 86)	Non-head resections (*n* = 96)	Head resections (*n* = 172)	Total (*n* = 511)
Death	0 (0%)	0 (0%)	2 (4.7%)	3 (3.5%)	2 (2.1%)	4 (2.3%)	11 (2.2%)
Wound infection	1 (1.5%)	4 (8.2%)	2 (4.7%)	16 (18.6%)	6 (6.3%)	17 (9.9%)	46 (9%)
Pancreatic fistula	3 (4.5%)	4 (8.2%)	0 (0%)	14 (16.3%)	5 (5.2%)	14 (8.2%)	40 (7.8%)
Gastroparesis	1 (1.5%)	5 (10.2%)	1 (2.3%)	10 (11.6%)	1 (1%)	16 (9.4%)	34 (6.7%)
Intraabdominal abscess	3 (4.5%)	6 (12.2%)	3 (7%)	16 (18.6%)	5 (5.2%)	20 (11.7%)	53 (10.4%)
Bile leak	0 (0%)	3 (6.1%)	0 (0%)	1 (1.2%)	0 (0%)	1 (0.6%)	5 (1%)
Pneumonia	0 (0%)	2 (4.1%)	2 (4.7%)	6 (7%)	2 (2.1%)	7 (4.1%)	19 (3.7%)
Urinary tract infection	1 (1.5%)	1 (2%)	2 (4.7%)	2 (2.3%)	5 (5.2%)	14 (8.2%)	25 (4.9%)
Postoperative bleeding	2 (3%)	1 (2%)	0 (0%)	4 (4.7%)	1 (1%)	4 (2.3%)	12 (2.3%)
Reoperation	3 (4.5%)	3 (6.1%)	4 (9.3%)	7 (8.1%)	1 (1%)	13 (7.6%)	31 (6.1%)
Other	9 (13.6%)	18 (36.7%)	8 (18.6)	30 (34.9%)	14 (14.6%)	65 (38%)	144 (28.2%)

*Each study year was listed by number of resections performed. The years were then divided by volume into three roughly equally sized groups: high, medium, and low.

**Table 3:** Postoperative complication and mortality rates by low, medium, and low volume years as defined by the number of *non-head resections* performed.*

	Low volume years		Medium volume years		High volume years		
	Non-head resections (*n* = 42)	Head resections (*n* = 93)	Non-head resections (*n* = 57)	Head resections (*n* = 64)	Non-head resections (*n* = 106)	Head resections (*n* = 149)	Total (*n* = 511)
Death	1 (2.1%)	3 (3.2%)	1 (1.8%)	1 (1.6%)	2 (1.9%)	3 (2%)	11 (2.2%)
Wound infection	2 (4.8%)	14 (15.1%)	1 (1.8%)	10 (15.6%)	6 (5.7%)	13 (8.7%)	46 (9%)
Pancreatic fistula	1 (2.4%)	9 (9.7%)	1 (1.8%)	9 (14.1%)	6 (5.7%)	14 (9.4%)	40 (7.8%)
Gastroparesis	1 (2.4%)	6 (6.5%)	1 (1.8%)	9 (14.1%)	1 (0.9%)	16 (10.7%)	34 (6.7%)
Intraabdominal abscess	1 (2.4%)	14 (15.1%)	4 (7%)	10 (15.6%)	6 (5.7%)	18 (12.1%)	53 (10.4%)
Bile leak	0 (0%)	2 (2.2%)	0 (0%)	1 (1.6%)	0 (0%)	2 (1.3%)	5 (1%)
Pneumonia	1 (2.4%)	7 (7.5%)	1 (1.8%)	3 (4.7%)	2 (1.9%)	5 (3.4%)	19 (3.7%)
Urinary tract infection	0 (0%)	6 (6.5%)	3 (5.3%)	2 (3.1%)	5 (4.7%)	9 (6%)	25 (4.9%)
Postoperative bleeding	0 (0%)	6 (96.5%)	1 (1.8%)	0 (0%)	2 (1.9%)	3 (2%)	12 (2.3%)
Reoperation	3 (7.1%)	7 (97.5%)	3 (5.3%)	6 (9.4%)	2 (1.9%)	10 (6.7%)	31 (6.1%)
Other	7 (16.7%)	38 (40.9%)	9 (15.8%)	22 (34.4%)	15 (14.2%)	53 (35.6%)	144 (28.2%)

*Each study year was listed by number of resections performed. The years were then divided by volume into three roughly equally sized groups: high, medium, and low.
